# The effects of endocrine disruptors on the female reproductive system

**DOI:** 10.55730/1300-0144.6125

**Published:** 2025-10-09

**Authors:** Rıza Gökhan BAYKAL, Reyhan ERSOY

**Affiliations:** 1 Department of Endocrinology and Metabolism, Ankara Bilkent City Hospital, Ankara, Turkiye; 2 Division of Endocrinology and Metabolism, Department of Internal Medicine, Ankara Yıldırım Beyazıt University, Ankara, Turkiye

**Keywords:** Endocrine disruptors, female reproductive system, infertility, bisphenol A, microplastics, nanoplastics

## Abstract

Endocrine-disrupting chemicals (EDCs) are a diverse, comprehensive group of mostly synthetic chemicals that disrupt many physiological functions in humans and animals. EDCs are particularly disruptive to the female reproductive system. Reproductive function in women is a dynamic process regulated by the hypothalamic–pituitary–ovarian axis. EDCs show their effects on the reproductive system through estrogenic, antiestrogenic, androgenic, and antiandrogenic effects or by directly affecting gonadotropin-releasing hormone secretion. Disruption in the menstrual cycle, decrease in fertility, infertility, increased risk of miscarriage, polycystic ovary syndrome, endometriosis, early or delayed puberty, and hormone-sensitive cancers can be listed as the main negative effects of endocrine disruptors on the female reproductive system. In this review, findings on the effects of the most studied EDCs, bisphenol A, phthalates, methoxychlor ethane, tetrachlorodibenzo-p-dioxin, atrazine, per- and polyfluoroalkyl substances, and micro- and nanoplastics on the female reproductive system are summarized.

## 1. Introduction

The term “endocrine disrupting chemicals (EDCs)” was officially introduced in 1991 at the Wingspread Conference Center in Wisconsin [1]. EDCs are exogenous substances that disrupt hormones involved in homeostasis, reproduction, and development through their synthesis, secretion, transport, signaling, or metabolism. They have been detected in adipose tissue, urine, amniotic fluid, and blood. They are found in products such as flame retardants, braking fluids, and adhesives. They are used in the production of plastic food boxes, polyvinyl chloride, pacifiers, medicines, cosmetics, hydraulic fluids, printing inks, receipts, and raincoats. They are also a component of cleaning agents, air fresheners, and polyvinyl products. They can also be present in soil or plants, as well as in the smoke from burning wood. Our diet contains EDCs, which are present in vegetables, green tea, fruits, and chocolate. Some of the commonly found EDCs in the environment are shown in Table [2].

According to a 2007 theory by Louis and Cooney, “environmental factors” may interfere with the development of the female reproductive system, resulting in decreased fertility. Studies showed that EDCs have a role in a variety of gynecologic diseases and disorders such as uterine dysfunction, fallopian tubal dysfunction, urogenital abnormalities, germline abnormalities, transgenerational defects in the offspring, premature or delayed puberty, infertility, polycystic ovary syndrome (PCOS), and endometriosis, and even cause uterine and ovarian cancer. Because EDCs are typically manufactured with long half-lives to benefit the industry, they do not degrade quickly and instead accumulate in the bodies of almost all humans and animals [2–4].

A large proportion of the population is regularly exposed to several endocrine disruptors; however, their effects become apparent only later in life. Additionally, in some circumstances, these effects may last for several generations and be inherited [5]. In both female and male rodents, EDCs have been shown to have transgenerational and multigenerational effects on reproduction. Uncertainty surrounds the mechanisms by which these substances affect reproduction, particularly in a transgenerational manner. To fully comprehend how these substances affect reproduction and how those effects span several generations, more research is needed [6–8].

The female reproductive system is generally controlled by luteinizing hormone (LH) and follicle-stimulating hormone (FSH) secreted by the anterior pituitary gland. EDCs exert their actions through various receptors, including nuclear receptors, neurotransmitter receptors, or orphan receptors. They have agonistic or antagonistic effects when hormones bind to their receptors. Epidemiological studies have demonstrated that exposure to perfluoroalkyls, polyfluoroalkyl compounds, polychlorinated biphenyls, and phthalates may cause irregularity of menstrual cycles, reduced ovarian reserve, reduced fertility, earlier onset of menopause, and premature ovarian insufficiency. Some EDCs, including bisphenol A (BPA), parabens, phthalates, and polychlorinated biphenyls, have been shown to cause follicle loss in experimental tests. The underlying mechanisms may include altered receptor-mediated proapoptotic pathways, signal transduction, cell cycle arrest, and epigenetic change, which may result in decreased ovarian follicular development [9].

## 2. Effects of endocrine disruptors

### 2.1. BPA

BPA is an estrogenic monomer [10]. Although it was the first to be synthesized, evidence gathered in 1936 showed that it bound preferentially to the nuclear estrogen receptor and had limited estrogenic activity. Its effects depend on the dosage, the target tissue, and the stage of tissue development at the site of action [11]. Depending on the tissue they target and the receptors they affect, estrogenic or antiestrogenic actions may occur [12].

BPA has been detected in ovarian follicular fluids. High exposure to BPA was associated with lower peak estradiol (E2) in response to human chorionic gonadotropin hyperstimulation, fewer fertilized oocytes, fewer mature oocytes, a lower probability of fertilization, increased implantation failure, and infertility [13–18]. BPA can cause infertility because of its harmful effects on follicle development [19,20].

In addition to BPA, phthalates, tetrachlorodibenzo-p-dioxin (TCDD), and methoxychlor ethane (MXC) also negatively impact follicle growth [21]. Exposure to BPA resulted in a significant decrease in blood estradiol (E2) levels, leading to enhanced luteal regression and follicular atresia due to elevated caspase-3-mediated cell death in ovarian cells [22].

Reduced antral follicle counts and diminished oocyte quantities have been associated with heightened BPA levels in women undergoing reproductive therapies. Exposure to BPA has been suggested to accelerate ovarian failure [23]. In a study of 137 women undergoing in vitro fertilization, a relationship between BPA levels and unsuccessful implantation was observed [13]. In a different study, the levels of BPA in infertile women were shown to be greater than those in fertile women [18]. According to a study conducted on mice, exposure to BPA during pregnancy impacts the first generation’s ability to reproduce, with inevitable consequences potentially lasting throughout generations [24].

Research indicates that BPA concentrations are elevated in women with PCOS compared to those who are healthy. Moreover, a positive correlation between insulin resistance and serum BPA concentration was observed in women with PCOS [25–27].

Furthermore, an association was found between body mass index, total testosterone, free testosterone, dehydroepiandrosterone, and dehydroepiandrosterone sulfate (with and without PCOS) and BPA. However, no correlation between serum BPA and any other sex hormones, LH, FSH, or E2, was confirmed [19,28].

It has been demonstrated that BPA crosses the placental barrier and has been detected in the fetus (serum) and in the amniotic fluid as a potential hazard. The amniotic fluid in the middle of pregnancy had the highest levels of BPA, which is subsequently eliminated when the fetal liver matures [29,30].

In an occupational case–control study, prolactin concentration was significantly higher in BPA-exposed women vs. nonexposed women [31].

### 2.2. Phthalates

Phthalates and related esters are a broad class of chemicals widely used in the cosmetic, plastic, coating, toy, and medical supply industries, as well as in the production of syringes, blood bags, and other medical supplies. Three esters are considered endocrine disruptors with estrogenic effects: benzyl butyl phthalate, dibutyl phthalate, and diethylhexyl phthalate (DEHP) [11].

Research indicates that urinary phthalate levels were greater in 56 couples who applied for assisted reproductive procedures than in couples who had one or more children through spontaneous pregnancy [32]. Rats exposed to 600 mg/kg of DEHP for 60 consecutive days exhibited an increase in atretic follicles and a decrease in the numbers of primary and secondary follicles [33].

Phthalates may exert a proliferative influence on endometrial tissue. A case–control study revealed that women with endometriosis exhibited markedly elevated levels of mono-n-butyl-phthalates in their urine compared to the control group [34]. According to another study, levels of six phthalate metabolites are twice as high in women with pelvic endometriosis as in controls [35]. The mechanism by which phthalates cause endometriosis is unclear. To date, only Kim et al. have demonstrated in an in vitro study that DEHP stimulates endometrial stromal cells and increases Ishikawa cell viability [36].

The National Health and Nutrition Examination Survey (NHANES) study from 2010 revealed that 1227 women had a higher risk of myoma when exposed to monobenzyl phthalate. On the other hand, there was an inverse correlation found between the beginning of disease and various phthalates, including mono-(2-ethyl-oxohexyl) phthalate, mono-(2-ethyl-5-hydrohexyl) phthalate, and mono-(2-ethylhexyl) phthalate [37].

Limited research has shown irregularities in menstrual periods resulting from phthalate exposure [11].

### 2.3. MXC

Methoxychlor [1,1,1-trichloro-2,2-bis(4-methoxyphenyl)ethane] is a chlorinated hydrocarbon pesticide frequently utilized in the United States as a substitute for 1,1,1-trichloro-2,2-bis(p-chlorophenyl)ethane (DDT). Although MXC is a weak estrogenic chemical, its principal metabolite, 2,2-bis(p-hydroxyphenyl)-1,1,1-trichloroethane, exhibits estrogenic, antiestrogenic, or antiandrogenic effects depending upon the receptor subtype it engages. MXC impedes folliculogenesis in vivo by elevating the expression of anti-Müllerian hormone in preantral and early antral follicles [38].

### 2.4. TCDD

The 2,3,7,8-tetrachlorodibenzo-p-dioxin is the most toxic congener of a group of halogenated dioxins that persist in the environment as toxic pollutants [39]. TCDD exhibits antiproliferative effects on the rat ovaries and can disrupt ovarian steroidogenesis [40,41]. In female Rhesus monkeys, TCDD may cause peritoneal endometriosis; the extent of the effect is dependent on the contamination level [42]. As demonstrated by Bredhult, TCCD’s estrogenic activity induces angiogenesis, leading to endometrial cell proliferation [43].

### 2.5. Atrazine

Atrazine (2-chloro-4-ethylamino-6-isopropylamino-1,3,5-s-triazine), a chlorotriazine, is predominantly utilized in agriculture as a herbicide. Pesticides and their metabolites contaminate drinking water sources and persist for long periods [44]. The effects of atrazine on steroidogenesis appear to vary with age, dose, and kind of experiment. In vitro research revealed that atrazine may change the expression of several enzymes involved in steroidogenesis and the levels of E2 in immature rat female granulosa cells [45]. Repeated dosages of atrazine enhanced steroidogenic enzyme expression and levels of sex hormones, as shown in in vivo investigations in adult animals [46–48]. Studies including female patients have not been conducted in adequate numbers. To confirm if these pesticides are detrimental to steroidogenesis and fertility, more investigation is required.

### 2.6. Micro- and nanoplastics

Currently, one of the most important issues in global health is plastic pollution. Both people and animals consume micro- and nanoplastics (MNPs), primarily through food and drink, and they can cross important epithelial barriers. Plastic contamination has been found in fetal fluid, infant meconium, and human placentas, suggesting that plastics are present from the moment of birth. Studies examining the effects of MNP exposure during the periconception and embryonic stages are currently scarce, despite this being a highly sensitive period that warrants careful consideration, given the increasing prevalence of plastics in our environment [49]. The majority of the data now available to us on the possible effects of MNPs on reproduction comes from recent studies on rodents and on aquatic and soil fauna [50].

MNPs accumulate in uterine tissue and several ovarian compartments, including developing follicles, in both rat and mouse models after oral exposure [51]. There are discernible alterations in reproductive hormone signaling, characterized by decreased levels of anti-Mullerian hormone and E2 in the bloodstream, and elevated levels of testosterone, FSH, and LH [52–54]. It is believed that oxidative stress and the accumulation of MNPs in female reproductive organs promote the proliferation of excess fibroblasts and the development of fibrosis [55,56]. MNPs were also found in the follicular fluid of patients undergoing fertility treatment [57]. These plastic particles most likely have additional negative impacts on oocyte health and offspring health, partly due to altered oocyte epigenetic imprinting [51].

### 2.7. Per- and polyfluoroalkyl substances

Per- and polyfluoroalkyl substances (PFAS) are a large family of synthetic fluorinated compounds widely used in industrial applications and consumer products, including nonstick cookware, water- and stain-resistant textiles, food packaging, and firefighting foams. Due to their high chemical stability and environmental persistence, PFAS are often referred to as “forever chemicals” and bioaccumulate in human tissues. In recent years, PFAS have been increasingly recognized as emerging EDCs that may adversely affect female reproductive health [58].

Experimental studies indicate that PFAS can interfere with the hypothalamic–pituitary–ovarian axis, alter gonadotropin and sex-steroid secretion, and impair ovarian function. Proposed mechanisms include disruption of nuclear receptor signaling, mitochondrial dysfunction, oxidative stress, impaired gap-junction communication, and epigenetic modifications within ovarian and endometrial cells. These changes have been linked to disturbances in folliculogenesis, altered steroidogenesis, and increased risk of reproductive endocrine disorders such as primary ovarian insufficiency, diminished ovarian reserve, polycystic ovary syndrome, and endometriosis [59,60].

Epidemiological evidence suggests that higher PFAS exposure is associated with reduced fecundability, prolonged time-to-pregnancy, and altered response to controlled ovarian stimulation in women undergoing assisted reproductive techniques. Several cohort and case–control studies have reported associations between serum or follicular-fluid PFAS concentrations and decreased markers of ovarian reserve, reduced embryo quality, and an increased risk of adverse pregnancy outcomes, including sporadic and recurrent miscarriage, although results are not entirely consistent across all PFAS congeners and study populations [61–63].

In addition, PFAS exposure has been linked to accelerated ovarian aging and earlier natural menopause. Prospective cohort data in midlife women indicate that higher cumulative PFAS burdens are associated with a shorter time to natural menopause and a higher odds of early or premature menopause, suggesting a potential role for these compounds in the pathogenesis of primary ovarian insufficiency and the age-related decline in reproductive capacity [64,65].

## 3. Conclusion

Understanding the intricate interactions between environmental factors and health outcomes requires examining EDCs in relation to women’s health. It enables to creation of risk-reduction plans, defends reproductive and general health, and provides information for public policy decisions that preserve women’s welfare. When assessing patients, healthcare providers need to be aware of the potential risks associated with EDC exposure and inquire about environmental exposures. This could lead to more accurate diagnoses and customized treatment plans.

## Figures and Tables

**Table:** Major endocrine disruptors.

Polychlorinated biphenyls (PCBs)
Polybrominated biphenyls (PBBs)
2,3,7,8-tetrachlorodibenzo p-dioxin (TCDD)
Plastics such as bisphenol A (BPA)
Plasticizers such as phthalates
Pesticides such as methoxychlor, chlorpyrifos, dichlorodiphenyl trichloroethane (DDT)
Fungicides such as vinclozolin
Pharmaceutical compounds such as diethylstilbestrol (DES)
Micro and nanoplastics
Per- and polyfluoroalkyl substances (PFAS)
